# Spatiotemporal fluctuations in fluorescence intensity of rhodamine phalloidin–labeled actin filaments

**DOI:** 10.1016/j.jbc.2025.110417

**Published:** 2025-06-24

**Authors:** Kenta Toshino, Yosuke Yamazaki, Shunsuke Ando, Ryuichi Kaneda, Kazunori Ono, Takahiro Suzuki, Saku T. Kijima, Taro Q.P. Uyeda

**Affiliations:** 1 Department of Pure and Applied Physics, Graduate School of Advanced Science and Engineering, Waseda University, Shinjuku, Tokyo, Japan; 2 Biomedical Research Institute, National Institute of Advanced Industrial Science and Technology, Ibaraki, Japan

**Keywords:** actin, fluorescence, cytoskeleton, protein structure, microscopy

## Abstract

Phalloidin (Ph) is widely used for fluorescent labeling of actin filaments. We observed ADP–actin filaments labeled with rhodamine phalloidin (RhPh) or Alexa488–Ph *in vitro* and discovered that the fluorescence intensities along the filaments showed a mottled pattern of bright and dark regions. Filaments labeled with substoichiometric RhPh exhibited more significant fluorescence inhomogeneities than those labeled with excess RhPh. Because the quantum yield of Alexa488 fluorescence is hardly affected by the environment, we concluded that the inhomogeneities arise from nonuniform Ph binding density rather than locally inhomogeneous quantum yield of the fluorophores. Simulations assuming random RhPh binding alone partially produced fluorescence inhomogeneities, but the degree of inhomogeneities was significantly smaller than the experimental results. Furthermore, filaments colabeled with RhPh and Alexa488–Ph showed a positive correlation in fluorescence intensities of Rh and Alexa488. Moreover, addition of inorganic phosphate suppressed the fluorescence inhomogeneities and the correlation between the Rh and Alexa488 fluorescence intensities. These results indicated that two mechanisms contribute to the nonuniform binding density of Ph: (i) stochastic binding and (ii) local differences in Ph binding affinity caused by inorganic phosphate–sensitive structural polymorphism of actin filaments. This structural polymorphism may also affect the binding of various actin-binding proteins, contributing to the functional differentiation of actin filaments *in vivo*. Moreover, those mottled fluorescence patterns dynamically fluctuated over time. These temporal fluorescence fluctuations required glucose and glucose oxidase but were suppressed by Trolox, likely reflecting photophysical properties of fluorophores influenced by oxygen scavengers and triplet-state quenchers. Taken together, we provide new insights into the structural polymorphism of actin filaments.

Actin filaments play central roles in many cellular functions, such as cell movement, cell division, and transcriptional regulation, depending on interactions with specific actin-binding proteins (ABPs) (1). In cells that exhibit amoeboid movement, interactions between ABPs such as cofilin and the Arp2/3 complex along the leading edge promote the formation of lamellipodia and facilitate the polymerization and depolymerization cycles of actin filaments, pushing the cell membrane forward (2, 3, 4). On the other hand, in the posterior cortex of those cells, actin filaments interact with the molecular motor myosin II to generate contractile forces through the hydrolysis of ATP, driving contraction of the rear end (5, 6). In this way, actin filaments perform diverse functions by selectively interacting with various ABPs locally within the cell. We have been investigating the influence of the structural polymorphism of actin filaments on the regulation of interactions with ABPs (7). When specific ABPs bind to actin filaments, structural changes propagate to the adjacent actin protomers. For example, when cofilin binds tightly to actin filaments and forms clusters, it changes the structure of bound actin protomers, accompanied by shortening of the helical pitch by approximately 25% (8, 9, 10). Notably, structural changes propagate to neighboring bare actin filament regions (11, 12, 13), suggesting that the increased binding affinity between bare actin filament regions around the cofilin clusters because of the propagation of the structural changes may extend the cofilin clusters. In addition, the formation and extension of clusters along actin filaments have been observed with other ABPs (14, 15, 16, 17, 18), and ABPs sparsely bound to actin filaments also alter the binding affinity of neighboring bare actin protomers to ABPs (17, 19). Furthermore, the structure of actin protomers naturally fluctuates (20, 21, 22). The release of inorganic phosphate (Pi) from actin filaments increases bending flexibility (23) and the fluctuations in actin filament twisting, resulting in a significantly higher fraction of supertwisted filaments (24). Cofilin preferentially binds to ADP–actin over ADP–Pi–actin (25, 26), possibly because of increased fluctuations in actin filament twisting caused by the release of Pi. The structural polymorphism of actin filaments, as discussed here, might contribute to selective ABP binding and play crucial roles in the functional differentiation of actin filaments *in vivo* (7, 27).

Phalloidin (Ph) is an actin-binding bicyclic peptide composed of seven amino acids isolated from the mushroom *Amanita phalloides*. While it binds tightly to actin filaments in a 1:1 M ratio with actin protomers, it does not bind to actin monomers (28). Ph binds between three adjacent actin protomers in a filament and positions itself between two protofilaments, thereby stabilizing interactions both vertically between two adjoining actin protomers in a protofilament and between the two protofilaments (29, 30, 31, 32). Fluorescent derivatives of Ph are widely used as probes to visualize actin filaments *in vivo* and *in vitro*. In particular, rhodamine (Rh) fluorophore significantly changes its fluorescence quantum yield depending on its surrounding environment (33, 34, 35, 36). Because of this property of Rh, rhodamine phalloidin (RhPh) displays higher fluorescence intensity when bound to actin filaments and surrounded by three actin protomers. RhPh is therefore frequently used for fluorescent observations of actin filaments (37, 38).

However, in this study, we found that the fluorescence intensity of actin filaments labeled with RhPh *in vitro* is not homogeneous along the filament length and also fluctuates over time. Spatial inhomogeneities were prominent when actin filaments were labeled with a substoichiometric amount of RhPh. Since the fluorescence quantum yield of Rh is sensitive to changes in the surrounding environment, we speculated that the dynamic structural polymorphism of actin filaments involving rearrangements of the Ph-binding site (39, 40) contributes to the temporal and spatial changes in fluorescence intensity of bound RhPh. In other words, RhPh staining may not accurately reflect the density of actin filaments in cell staining images but can be used to monitor the conformational dynamics of actin filaments in real time. Therefore, we studied those fluctuations in detail. Here, we report that the spatial inhomogeneity of fluorescence intensity depends on the nonuniform binding density of Ph, partly because of the structural polymorphism of actin filaments. However, the temporal fluctuation depends on glucose and glucose oxidase added to the observation solution as antibleaching reagents, suggesting that it reflects well-known photophysical properties of fluorophores influenced by oxygen scavengers, rather than the structural polymorphism of actin filaments.

## Results

### Spatial inhomogeneities in fluorescence intensity of actin filaments labeled with a substoichiometric concentration of fluorescent Ph

**Figure 1:**
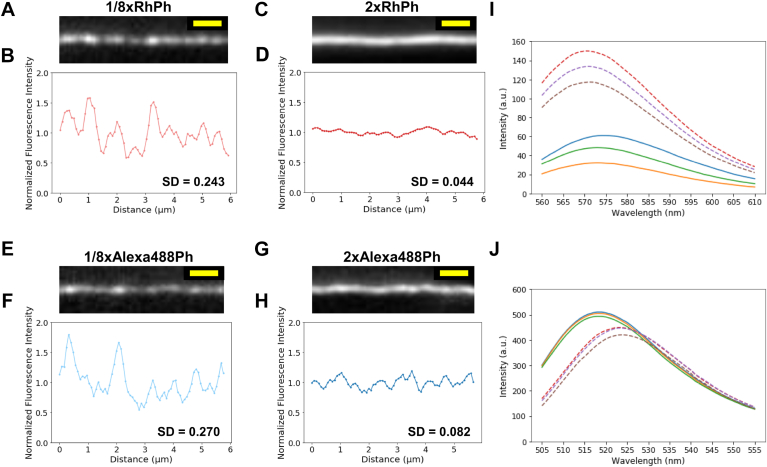
**Spatial fluorescence intensity inhomogeneities in ADP–actin filaments labeled with RhPh or Alexa488Ph.***A*, *C*, *E*, and *G*, representative fluorescence images of 1/8xRhPh–actin, 2xRhPh–actin, 1/8xAlexa488Ph–actin, and 2xAlexa488Ph–actin filaments, respectively, observed in F-buffer (without Trolox, glucose oxidase, or glucose) after linearization. Scale bars (*yellow*) represent 1 μm. *B*, *D*, *F*, and *H*, normalized fluorescence intensity distributions along the filaments shown in *A*, *C*, *E*, and *G*, respectively, with the mean fluorescence intensity along the filament set to 1. The SD of the fluorescence intensity along the length of the filament is shown in the *bottom right*. *I* and *J*, fluorescence spectra of 1 μM Rh-NHS (*I*) and 1 μM Alexa488-NHS (*J*) in pure water and methanol. The *solid lines* show three measurements in pure water, whereas the *dashed lines* show three measurements in methanol. The excitation light was 545 nm for Rh and 494 nm for Alexa488. Alexa488Ph, Alexa488–Ph; RhPh, rhodamine phalloidin.

First, 10 μM rabbit skeletal muscle G-actin was polymerized in F-buffer for 1 h at 23 °C. Most of the actin protomers in the resultant filaments should be in the ADP-bound state because of ATP hydrolysis and subsequent phosphate release during polymerization and aging (41, 42). Actin filaments were then diluted to various concentrations and labeled by incubating with 1 μM RhPh for 15 min at 23 °C. Actin filaments were immobilized onto an amino-silanized glass surface and observed under a fluorescence microscope. While antibleaching reagents, such as Trolox or a mixture of glucose, glucose oxidase, and catalase, are often used to suppress photobleaching during fluorescence microscopy, they were not used in this observation. The fluorescence images of actin filaments labeled with one-eighth of the molar ratio of RhPh (1/8xRhPh–actin filaments) revealed a submicron-scale mottled pattern of alternating bright and dark regions (Fig. 1, *A* and *B*). The fluorescence intensity of 100 filaments with a length of 2 μm or longer was 1031 ± 204 a.u. (mean ± SD). To quantitatively compare the spatial inhomogeneity of fluorescence intensity among populations of differently labeled actin filaments, the fluorescence intensity along each filament was normalized such that the average value was one for the filament, and then SD of the normalized fluorescence intensities was calculated for each filament. Subsequently, average and SD of these SDs were calculated for a set of 10 to 100 filaments. For 1/8xRhPh–actin filaments, these values were 0.242 ± 0.046. In contrast, actin filaments labeled with twice the molar ratio of RhPh (2xRhPh–actin filaments) exhibited relatively uniform fluorescence intensity along their lengths (Fig. 1, *C* and *D*). The fluorescence intensity along 2xRhPh–actin filaments was 9081 ± 852 a.u. (n = 96 filaments). When normalized as aforementioned, the average and SD of the SDs of fluorescence intensities along the length of the 96 filaments were 0.045 ± 0.010, which was significantly smaller than the 1/8RhPh–actin filaments (*p* < 0.001 by Welch's *t* test). The smaller average of SDs for the 2xRhPh–actin filaments compared with the 1/8xRhPh–actin filaments is consistent with the visual impression of the fluorescence images, and the average of SDs will serve as the quantitative index of the fluorescence intensity inhomogeneities in the subsequent analyses.

Given that the fluorescence properties of Rh are strongly sensitive to its environment (33, 34, 35, 36), the spatial inhomogeneity in fluorescence intensity may be attributed to two potential factors: fluctuations in the fluorescence quantum yield of Rh because of differences in the molecular structure of actin and nonuniform binding density of RhPh along the lengths of the filaments. To investigate the possible involvement of the properties of Rh, similar fluorescence observations were performed using a different fluorescent Ph, Alexa488–Ph (Alexa488Ph). Actin filaments labeled with one-eighth of the molar ratio of Alexa488Ph (1/8xAlexa488Ph–actin filaments) also exhibited a mottled fluorescence pattern, similar to 1/8xRhPh–actin filaments (Fig. 1, *E* and *F*). The average and SD of SDs of normalized fluorescence intensity along the length of 1/8xAlexa488Ph-labeled actin filaments was 0.279 ± 0.066 (n = 10 filaments). Actin filaments labeled with twice the molar ratio of Alexa488Ph (2xAlexa488Ph–actin filaments) showed a more uniform fluorescence intensity along the filaments compared with 1/8xAlexa488Ph–actin filaments (Fig. 1, *G* and *H*). The average and SD of SDs of normalized fluorescence intensity along the length of 2xAlexa488Ph–actin filaments was 0.086 ± 0.022 (n = 10 filaments).

To investigate the environmental dependence of Rh and Alexa488 fluorescence intensity, we measured the fluorescence intensity of the fluorophores in pure water and methanol using a fluorometer (Fig. 1, *I* and *J*). The maximum fluorescence intensity of Rh in methanol was at 571 nm and 2.8-fold higher than that in pure water at 574 nm (Fig. 1*I*), confirming a previous report (43). In contrast, the maximum fluorescence intensity of Alexa488 in methanol was at 523 nm and 1.1-fold lower than that in pure water at 518 nm (Fig. 1*J*), demonstrating that it is insensitive to the environment, relative to Rh. We also compared the fluorescence intensities in microscopic images captured by an electron multiplying charge-coupled device camera through appropriate filter sets. The fluorescence intensity of Rh in the 575 to 610 nm transmission band of the emission filter used during Rh observation was 2.53 times higher in methanol than in pure water, whereas the fluorescence of Alexa488 in the 510 to 555 nm transmission band of the emission filter used during Alexa488 observation was 0.91 times higher in methanol than in pure water.

### Factors affecting spatial inhomogeneities of the fluorescence intensity of fluorescent Ph

**Figure 2:**
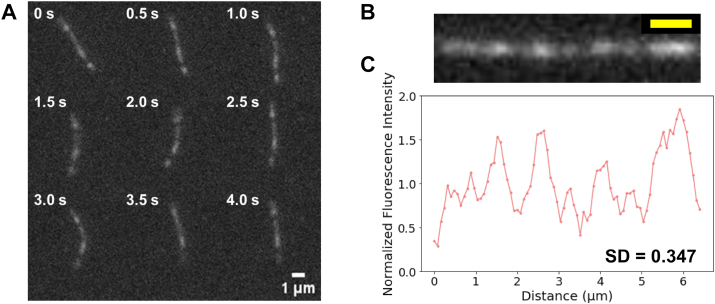
**Observation of 1/8xRhPh–actin filaments in F-buffer (without Trolox, glucose oxidase, or glucose) using a methylcellulose observation cell.***A*, fluorescence images of a 1/8xRhPh–actin filament taken at 0.5 s intervals. *B*, a fluorescence image of the1/8xRhPh–actin filament after linearization. Scale bar (in *yellow*) represents 1 μm. *C*, normalized fluorescence intensity distribution along the 1/8xRhPh–actin filament, with the mean fluorescence intensity along the filament set to 1. RhPh, rhodamine phalloidin.

In the aforementioned experiment, actin filaments were immobilized onto amino-silanized glass surfaces *via* electrostatic interactions. The amino groups of amino-silane might neutralize the negative charge on the surface of actin, potentially affecting the structure of actin protomers. Therefore, possible unevenness in the charge density of amino-silanized glass surfaces or uneven binding of the filament to the amino-silanized surface could contribute to spatial inhomogeneities in fluorescence intensity along the filaments. To address this possibility, we used a methylcellulose observation cell to observe 1/8xRhPh–actin filaments without immobilizing them to the glass surface. In the presence of the observation solution containing 0.4% methylcellulose, the actin filaments in the solution were pressed against the glass surface because of the excluded volume effect, which allows the observation of actin filaments that remain near the glass surface (44, 45). In this observation, while the actin filaments slowly moved on the surface, the fluorescence intensity along the filaments was obviously not homogeneous, and the bright and dark patterns along the fluorescence image of the filaments did not change over time (Fig. 2*A* and Video S1). The average and SD of SDs of normalized fluorescence intensity along the length of 1/8xRhPh–actin filaments observed using methylcellulose was 0.362 ± 0.083 (n = 10 filaments) (Fig. 2, *B* and *C*). The possibility that spatial fluorescence inhomogeneities are caused by denatured actin molecules incorporated into the filaments was ruled out by a control experiment using actin that underwent an additional polymerization–depolymerization cycle after thawing the frozen stock (Fig. S1).

**Figure 3:**
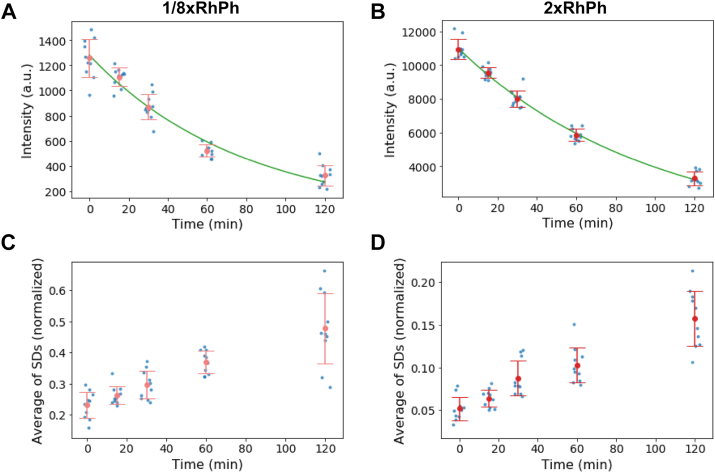
**Decrease in fluorescence intensity and increase in SD along the length of RhPh–actin filaments over time.** The imaging was performed at 0, 15, 30, 60, 90, and 120 min after flushing the flow cell with F-buffer containing antibleaching reagents (1 mM Trolox, 3 mg/ml glucose, 0.2 mg/ml glucose oxidase, and 0.04 mg/ml catalase). Ten actin filaments in a new imaging field of view were analyzed in each observation. *A* and *C*, 1/8xRhPh–actin filaments. *B* and *D*, 2xRhPh–actin filaments. *A* and *B*, a time-dependent decrease of the average fluorescence intensities ± SD of the two groups of RhPh–actin filaments. The *green lines* show fitting with a single exponential function. *C* and *D*, a time-dependent increase of the average ± SD of SDs of the normalized fluorescence intensities of each of the 10 RhPh–actin filaments. Each data point represents the intensity (*A* and *B*) and the SD (*C* and *D*) of fluorescence intensity along one actin filament in two sets of independent experiments. RhPh, rhodamine phalloidin.

In the experiments shown previously, free RhPh molecules not bound to actin filaments were washed out before observations by flushing F-buffer through the flow cell. Under this observation condition, new binding of RhPh was unlikely to occur, and bound RhPh would gradually dissociate from actin filaments over time. To investigate the effect of RhPh dissociation from actin filaments on the spatial inhomogeneity of fluorescence intensity, the decrease in fluorescence intensity of RhPh–actin filaments was followed by capturing fluorescence images at 0, 15, 30, 60, and 120 min after flushing the flow cell using F-buffer containing glucose, glucose oxidase, catalase, and Trolox to minimize photobleaching. Fluorescence intensity decreased for both 1/8xRhPh– and 2xRhPh–actin filaments (Fig. 3, *A* and *B*). These decreases were well fitted by single exponential decay, yielding time constants of 77 and 93 min, respectively. In addition, the average of SDs of normalized fluorescence intensity along the length increased over time (Fig. 3, *C* and *D*). The observations shown in Figures 1 and 2 were completed within 10 min after flushing out free RhPh in the flow cells, and the effects of RhPh dissociation on spatial inhomogeneity in fluorescence intensities should be negligible.

### Inhomogeneities in fluorescence intensity because of random binding of RhPh

**Figure 4:**
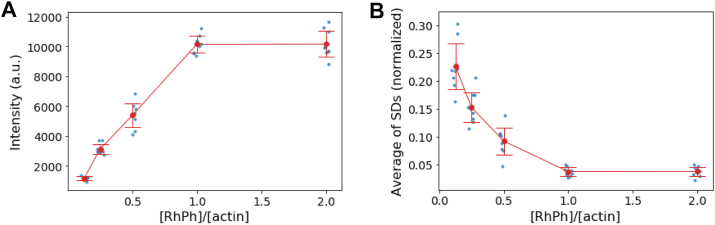
**Effects of labeling density on the average and SD of the fluorescence intensities along the length of RhPh–actin filaments.***A*, the average fluorescence intensity. *B*, the average of SDs of normalized fluorescence intensity along the length of filaments. In each observation, nine actin filaments were analyzed. Each data point represents the average fluorescence intensity (*A*) and the SD of normalized fluorescence intensity (*B*) along one actin filament in three independent experiments. RhPh, rhodamine phalloidin.

To determine the labeling density of 1/8xRhPh- and 2xRhPh-labeled actin filaments, the fluorescence intensity of filaments was investigated by incubating 1 μM actin filaments with increasing concentrations of RhPh until saturation (Fig. 4). To minimize the effects of RhPh dissociation, the filaments were observed within 1 min after washing the flow chamber during this experiment. As the concentration of RhPh increased, the fluorescence intensity along the filaments rose, whereas the average of SDs of normalized fluorescence intensities decreased. Saturation of fluorescence intensity was achieved at RhPh concentrations above 1 μM, with a saturated fluorescence intensity of 10,179 ± 891 a.u. (n = 9 filaments). Assuming that one RhPh molecule binds to each actin protomer at saturation, the labeling density of 1/8xRhPh- and 2xRhPh-labeled actin filaments was calculated as the ratio of their fluorescence intensity to the saturated fluorescence intensity. The average labeling densities were determined to be 10.1% for 1/8xRhPh-labeled filaments and 89.2% for 2xRhPh-labeled filaments.

The nonuniform fluorescence intensity in 1/8xAlexa488Ph–actin filaments and the lower SD observed in actin filaments labeled with excess RhPh indicate that the spatial inhomogeneities in fluorescence intensity are not because of fluctuations in the fluorescence quantum yield of Rh. This led us to conclude that the nonuniform binding density of Ph is the primary cause of the spatial inhomogeneities in fluorescence intensity. This nonuniform binding density of Ph may be attributed to two possible factors: (1) stochastic inhomogeneities in binding density because of random binding of Ph; (2) the spatial inhomogeneities in the structure of actin filaments leading to a nonuniform binding affinity for Ph. We simulated the random binding of Ph to investigate whether we could fully explain the observed spatial inhomogeneities in fluorescence intensity by stochastic inhomogeneities in binding density.

**Figure 5:**
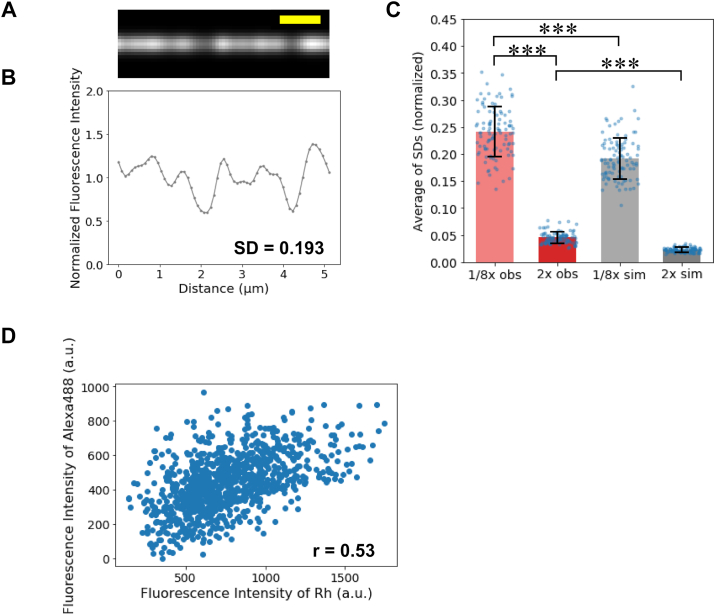
**Spatial inhomogeneities in fluorescence intensity because of random binding of RhPh.***A*, a representative simulated pseudo image of 10.1% binding density actin filaments. *B*, the fluorescence intensity distribution along the simulated fluorescence image. *C*, comparison of the average of SDs of the normalized observed fluorescence intensities along the 1/8xRhPh–actin filaments (n = 100 filaments), 2xRhPh–actin filaments (n = 96 filaments), and simulations at 10.1% binding density (n = 100 filaments) and 89.2% binding density (n = 100 filaments). ∗∗∗ indicates *p* < 0.001 (Welch’s *t* test). Each data point represents the SD of fluorescence intensity along one actin filament. The observed data were obtained by analyzing filaments in 25 images taken in five independent experiments. For the simulation data, 100 independent simulations were performed, and the SD calculated from each simulation was plotted as an individual data point. *D*, a scatter plot of the Rh and Alexa488 fluorescence intensities at each pixel in 1/8xRhPh–1/8xAlexa488Ph actin filaments. Total of 968 pixels along 15 actin filaments were analyzed. RhPh, rhodamine phalloidin.

In the simulation, we assumed that RhPh molecules bind to 2400 actin protomers arranged in one dimension with a certain probability. The number of RhPh molecules within 1 pixel (80 nm long) was calculated based on the average labeling density and the fact that there are about 32 actin protomers per pixel in the observed image. The fluorescence intensity of each pixel was calculated, considering the spatial spread of Rh fluorescence, which was determined from actual observations and computed using a Gaussian distribution. The simulation was performed for filaments labeled with 10.5% average labeling density (1/8xRhPh–actin filaments), where Rh molecules bind to each actin molecule with a probability of 10.5%. The simulated fluorescence intensity was not uniform (Fig. 5, *A* and *B*). The average and SD of SDs of fluorescence intensity calculated from this simulation for 1/8xRhPh–actin filaments was 0.192 ± 0.038 (n = 100 filaments), significantly smaller than the average of SDs of the observed fluorescence intensity (Fig. 5*C*). Similarly, simulations were conducted for 2xRhPh–actin filaments. Again, the average of SDs of fluorescence intensity was significantly smaller in the simulations compared with the observations. These results indicated that stochastic binding contributes to the observed spatial inhomogeneities of the RhPh fluorescence intensities. Still, they suggested that a part of spatial fluorescence inhomogeneity is due to spatial inhomogeneities in the structure of actin filaments.

To gain experimental support for this possibility, we simultaneously incubated actin filaments with 1/8xRhPh and 1/8xAlexa488Ph. Fluorescence intensities of Rh and Alexa488 were measured in 15 actin filaments, and the correlation coefficient was calculated by comparing the fluorescence intensities of Rh and Alexa488 in the same pixel (Fig. 5*D*). If each Ph bound randomly to actin, the correlation coefficient should be zero or negative because the two fluorescent Ph derivatives compete for the same binding site on actin. However, the actual correlation coefficient was 0.53. This positive correlation suggests that actin filaments contain regions where both RhPh and Alexa488Ph bind preferentially and regions where both avoid.

### Effects of bound Pi and divalent cations on spatial fluorescence inhomogeneity of RhPh–actin filaments

**Figure 6:**
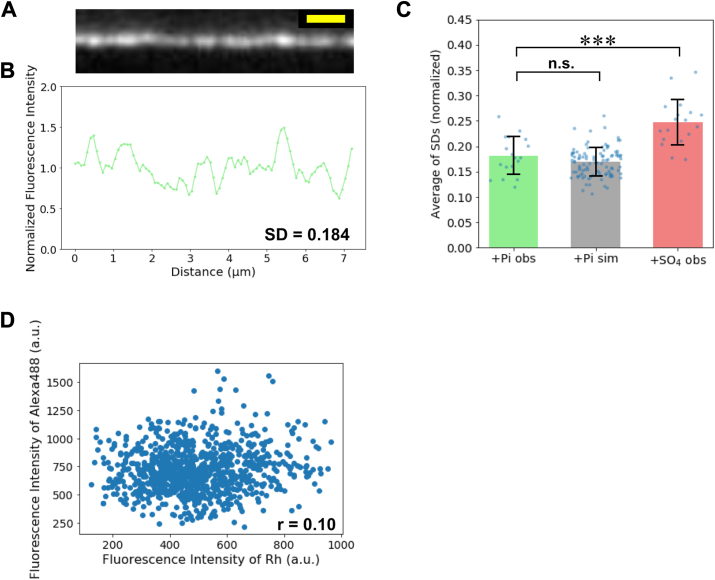
**Spatial inhomogeneity of fluorescence intensity in RhPh-labeled actin filaments in the presence of Pi.***A*, a representative fluorescence image of 1/8xRhPh–actin filaments labeled in the presence of 20 mM KH_2_PO_4_–K_2_HPO_4_ (ADP–Pi–actin). *B*, normalized fluorescence intensity distribution along the filament shown in *A*. *C*, comparison of the average of SDs of the normalized observed fluorescence intensities along 1/8xRhPh-labeled filaments under three conditions: ADP–actin + 20 mM KH_2_PO_4_–K_2_HPO_4_ (ADP–Pi, n = 18 filaments), ADP–actin + 20 mM K_2_SO_4_ (n = 18 filaments), and simulations at 13.4% binding density (n = 100 filaments). ∗∗∗ indicates *p* < 0.001 (Welch’s *t* test). Each point represents the SD of fluorescence intensity along one actin filament. The observed data were obtained by analyzing filaments in six images taken in two independent experiments. For the simulation data, 100 independent simulations were performed, and the SD calculated from each simulation was plotted as an individual data point. *D*, a scatter plot of the Rh and Alexa488 fluorescence intensities at each pixel in 1/8xRhPh–1/8xAlexa488Ph–actin filaments incubated in the presence of 20 mM KH_2_PO_4_–K_2_HPO_4_ (ADP–Pi–actin). Total of 888 pixels along nine actin filaments were analyzed. Pi, inorganic phosphate; RhPh, rhodamine phalloidin.

To investigate the possible effects of bound Pi on the inhomogeneous Ph binding affinity along actin filaments, we next examined filaments in the ADP–Pi state, in contrast to the ADP–actin filaments. Actin filaments (8 μM) were incubated for 10 min in the presence of 20 mM K_2_HPO_4_–KH_2_PO_4_ at pH 7.4 prior to RhPh labeling. According to a previous report, the equilibrium dissociation constant of Pi at pH 7.4 is approximately 2.5 mM (46). Therefore, the addition of 20 mM Pi is expected to shift the majority of actin protomers (∼90%) into the ADP–Pi state. Fluorescence intensity distributions were then analyzed as in the earlier part of this study (Fig. 6, *A* and *B*). The average fluorescence intensity of 1/8xRhPh-labeled filaments under this condition was 1365 ± 269 a.u. (n = 18 filaments), which corresponds to a labeling ratio of 13.4%. The average of SDs of the normalized fluorescence intensities was significantly lower, at 0.181 ± 0.037 (n = 18 filaments). This value closely matched the SD obtained from simulations, assuming random RhPh binding at the same 13.4% labeling ratio (Fig. 6*C*). To account for the potential effect of increased ionic strength because of 20 mM KH_2_PO_4_–K_2_HPO_4_ addition, we also analyzed actin filaments labeled with 1/8xRhPh in the presence of 20 mM K_2_SO_4_ as a control (Fig. S2, *A* and *B*). Under this condition, the SD of the normalized fluorescence intensities was 0.247 ± 0.045, which was significantly higher than that observed with KH_2_PO_4_–K_2_HPO_4_ and also exceeded the value obtained from simulations assuming random RhPh binding at the same labeling ratio (Figs. 6*C* and S2*C*). These results demonstrated that increased ionic strength had no effect on fluorescence inhomogeneity. Thus, the suppression observed with Pi reflects a Pi-specific effect. We propose that bound Pi may suppress nonrandom variations in Ph labeling. Supporting this idea, colabeling of ADP–Pi actin filaments with RhPh and Alexa488Ph resulted in a reduced correlation coefficient between the two fluorescence intensities (*r* = 0.10; Fig. 6*D*). In contrast, when ADP–actin filaments were colabeled in the presence of 20 mM K_2_SO_4_, the correlation coefficient remained high (*r* = 0.75; Fig. S2*D*), confirming that elevated ionic strength does not influence the spatial correlation of Ph labeling. These findings suggest that the presence of Pi reduces the heterogeneity of Ph affinity along actin filaments.

In the experiments described thus far, Ca^2+^-bound G-actin was polymerized in F-buffer containing Mg^2+^, so that the filaments were made of mixtures of Ca^2+^-bound and Mg^2+^-bound actin protomers. We therefore prepared Mg^2+^–actin filaments and Ca^2+^–actin filaments and examined the inhomogeneity of the fluorescence intensities when bound with 1/8 M ratio of fluorescent Ph. The two groups of filaments exhibited similar fluorescence intensity inhomogeneities, in terms of average of the SDs of normalized fluorescence intensities of RhPh and the correlation of RhPh and Alexa488Ph fluorescence intensities (Figs. S3 and S4).

### Temporal fluctuations in fluorescence intensity of RhPh–actin filaments

**Figure 7:**
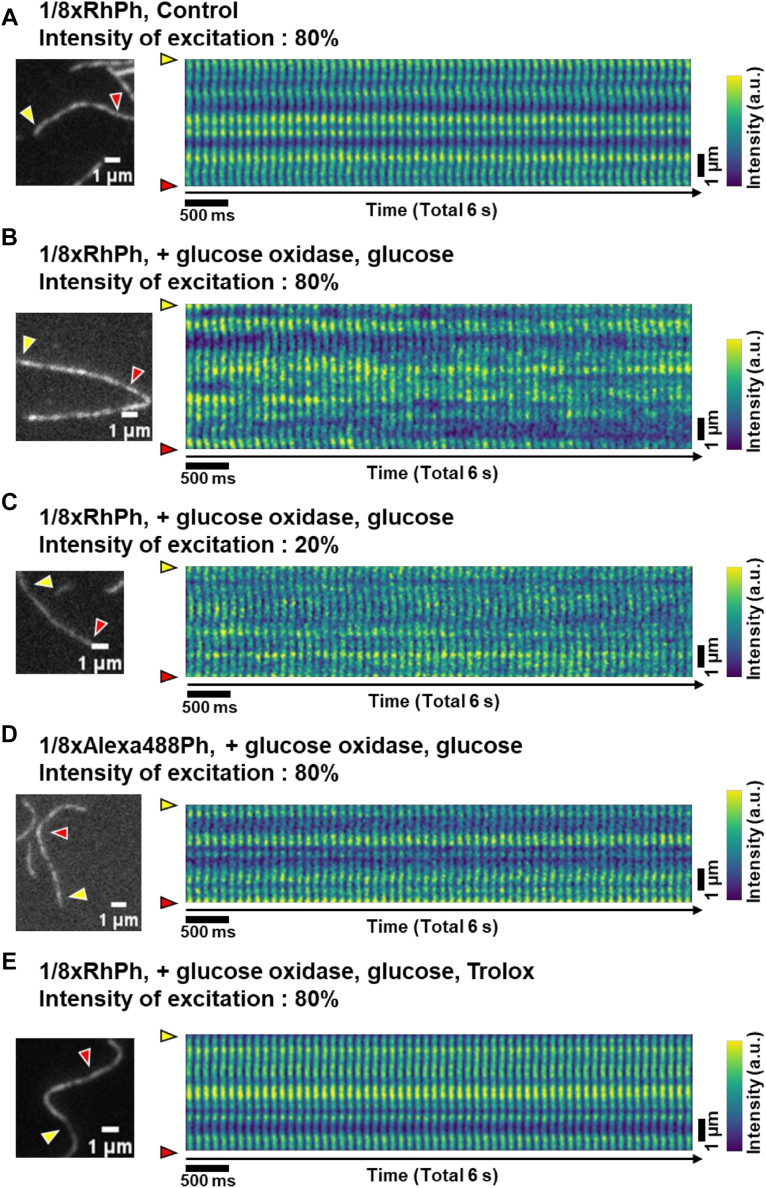
**Comparison of the temporal fluctuations in fluorescence intensity under different observation conditions.** The actin filaments were immobilized onto amino-silanized glass surface. Time-lapse observations were conducted with successive 100 ms exposures for 6 s. A snapshot fluorescence image of actin filaments during the time-lapse observation (*left*) and a kymograph of the linearized images between the *yellow* and *red arrows* of the filament in the snapshot (*right*) are shown. The fluorescence intensities of each pixel are color-coded as shown on the *right*, with *yellow* indicating higher values and *dark green* indicating lower values. *A*, 1/8xRhPh–actin filaments observed in F-buffer without any antibleaching reagents at 80% excitation light intensity. *B*, 1/8xRhPh–actin filaments observed in F-buffer containing 3 mg/ml glucose and 0.2 mg/ml glucose oxidase at 80% excitation light intensity. *C*, 1/8xRhPh–actin filaments observed in F-buffer containing 3 mg/ml glucose and 0.2 mg/ml glucose oxidase at 20% excitation light intensity. *D*, 1/8xAlexa488Ph–actin filaments observed in F-buffer containing 3 mg/ml glucose and 0.2 mg/ml glucose oxidase at 80% excitation light intensity. *E*, a 1/8ⅹRhP–actin filament in F-buffer containing 3 mg/ml glucose, 0.2 mg/ml glucose oxidase, and 1 mM Trolox at 80% excitation light intensity. RhPh, rhodamine phalloidin.

In addition to the spatial inhomogeneities in fluorescence intensity of RhPh–actin filaments, prominent temporal changes between bright and dark regions of the filaments over time were observed in F-buffer containing the antibleaching reagents (3 mg/ml glucose, 0.2 mg/ml glucose oxidase, and 0.04 mg/ml catalase) (Video S2). Surprisingly, however, in F-buffer without the antibleaching reagents, we detected virtually no temporal fluctuations in the fluorescence intensities (Fig. 7*A*). Therefore, we investigated which antibleaching reagents contributed to the temporal fluctuations. In F-buffer containing only glucose and glucose oxidase, temporal fluctuations in fluorescence intensity were observed (Fig. 7*B*). Furthermore, the temporal fluctuations in fluorescence intensity were more pronounced when stronger excitation light was applied (Fig. 7, *B* and *C*). Therefore, we concluded that Trolox and catalase do not contribute to the temporal fluctuations in fluorescence intensity of RhPh–actin filaments, and these fluctuations can be observed when intense excitation light is applied in the presence of glucose and glucose oxidase. No temporal fluctuations in fluorescence intensity were observed in the case of Alexa488Ph–actin filaments even when intense excitation light and F-buffer containing glucose and glucose oxidase were used (Fig. 7*D*).

### Suppression of temporal fluctuations by Trolox

Glucose and glucose oxidase are the components of the glucose oxidase–catalase antibleaching system commonly employed in fluorescence microscopy (38). These agents consume oxygen present in the observation solution and reduce photobleaching. Trolox, an analog of vitamin E, is another frequently used reagent for preventing photobleaching. Trolox quickly returns the excited triplet-state fluorescent molecules to their ground state, thus preventing photobleaching (47, 48, 49). Both systems individually exhibit antibleaching effects, and their combination has been reported to reduce the photobleaching rate more efficiently than either one system, while also effectively suppressing fluorophore blinking (50, 51, 52). When observed in a solution containing glucose, glucose oxidase, and Trolox, the temporal fluctuations in the fluorescence intensity of RhPh–actin filaments were no longer observed (Fig. 7*E*). In other words, the glucose/glucose oxidase–dependent temporal fluctuations in the fluorescence intensity of RhPh–actin filaments are suppressed by Trolox.

## Discussion

### Mechanism of spatial inhomogeneities in fluorescence intensity of RhPh–actin filaments

In this study, we quantitatively evaluated the extent of spatial inhomogeneities in fluorescence intensity by comparing the average of SDs of fluorescence intensity along the filaments and investigated the underlying mechanism. The submicron-scale spatial inhomogeneity in fluorescence intensity could be attributed to three possible mechanisms: (1) fluctuation in the fluorescence quantum yield of Rh because of differences in the structure of actin filaments; (2) nonuniform binding density of Ph because of nonuniform binding affinity of Ph resulting from differences in the structure of actin filaments; (3) nonuniform binding density of Ph resulting from the stochastic random binding of Ph at substoichiometric ratios to actin. If this inhomogeneity was attributed to local variation in the fluorescence quantum yield of the fluorophore owing to presumptive local variation in the atomic structure of actin molecules along the filament, then the spatial inhomogeneities would still occur, even when the actin filaments are densely labeled with RhPh. However, the extent of spatial inhomogeneities in the densely labeled 2xRhPh–actin filaments was much smaller than that of the sparsely labeled 1/8xRhPh–actin filaments (Fig. 1, *C* and *D*). Moreover, similar spatial inhomogeneities were observed when using Alexa488Ph instead of RhPh (Fig. 1, *E*–*H*), even though the fluorescence quantum yield of Alexa488 is less sensitive to local environments than Rh (Fig. 1, *I* and *J*). These results led us to conclude that fluctuation in fluorescence quantum yields did not contribute to the observed spatial inhomogeneities of the fluorescence intensity in actin filaments labeled with a substoichiometric amount of either RhPh or Alexa488Ph.

Thus, the spatial inhomogeneities in fluorescence intensity should reflect the inhomogeneous binding of RhPh to actin filaments. The nonuniform binding density of Ph may reflect differences in the structure of actin filaments. Alternatively, the nonuniform binding density may be caused simply by stochastic binding. Indeed, fluorescent tubulin is incorporated into microtubules stochastically, leading to the formation of speckled fluorescence patterns along filaments (53). To elucidate the contribution of the latter mechanism, we simulated fluorescence intensities along homogenous filaments at different average binding densities (Fig. 5, *A* and *B*). The simulations demonstrated that while random binding causes spatial inhomogeneities in fluorescence intensity, the extent of these inhomogeneities is significantly smaller than those observed experimentally (Fig. 5, *C* and *D*), in contrast to the case of microtubules mentioned above (53). Furthermore, when actin filaments were labeled simultaneously with substoichiometric amounts of RhPh and Alexa488Ph, the fluorescence intensities of Rh and Alexa488 at each pixel showed a positive correlation (Fig. 5*D*). These results strongly suggest the presence of micrometer-scaled actin filament regions in which a large number of neighboring actin protomers tend to assume the same high- or low-affinity state for Ph. Such cooperative behavior may arise from conformational fluctuations of the Ph-binding sites of actin protomers themselves. It has been proposed that these binding sites fluctuate between open and closed states (39, 40), which could directly affect the local affinity for Ph. If the Ph-binding sites of neighboring actin protomers transiently adopt the same conformational state in a coordinated manner, this could explain the observed spatial clustering of high and low Ph-binding densities along the filaments.

Compared with the ADP state, actin filaments in the ADP–Pi state exhibited a smaller spatial fluorescence inhomogeneity, which was comparable to that predicted by random binding simulations (Fig. 6*C*). In addition, the correlation coefficient between RhPh and Alexa488Ph fluorescence intensities was lower (0.10, Fig. 6*D*) than that of ADP–actin filaments (0.75, Fig. S2*D*). These findings suggest that actin filaments in the ADP–Pi state adopt a more structurally uniform conformation. Recent high-speed atomic force microscopic analysis has demonstrated that ADP–actin filaments exhibit greater fluctuations in half-helical pitch and are more prone to forming supertwisted helical structures than those in the ADP–Pi state (24). The spatial fluorescence fluctuations observed in ADP–actin filaments may reflect such enhanced helical pitch variability, whereas bound Pi may suppress these structural fluctuations, resulting in a more homogeneous distribution of RhPh fluorescence along the filaments. In addition, the variation in Ph binding affinity, previously suggested to arise from fluctuations in the conformational state of Ph-binding sites (39, 40), may also be attenuated in the ADP–Pi state. While it remains unclear whether either or both these structural fluctuation mechanisms contribute to the observed fluorescence uniformity, our findings raise the possibility that bound Pi promotes fluorescence homogeneity through the suppression of one or more underlying structural fluctuations.

In summary, the present study suggested that the cooperative spatial inhomogeneities in the structure of actin filaments result in locally different affinities for Ph. Although the nonuniform Ph binding to actin filaments may lack direct physiological significance, the underlying structural polymorphism of actin filaments could hold physiological relevance. Interestingly, our findings suggest that Pi binding may stabilize actin filaments in a more homogeneous structural conformation. This implies that the nucleotide state of actin not only governs filament dynamics such as polymerization and depolymerization but also modulates the conformational fluctuations and their spatial distribution within filaments, with coordinated structural variations occurring at the submicron scale, potentially influencing the binding behavior of ABPs more broadly. Therefore, it is critical to examine whether interactions with ABPs are correlated with high and low binding densities of Ph along actin filaments, by observing, for instance, the binding density of RhPh when added to actin filaments that have bound ABP clusters (14, 15). Further studies are also needed to reveal the structural identities of the high and low Ph affinity states of actin filaments, as well as to determine whether nucleotide- or ABP-induced modulation of filament structure contributes to the spatial patterning of ABP localization and the functional diversification of the actin cytoskeleton in cells.

### Mechanism of temporal fluctuations in fluorescence intensity of RhPh–actin filaments

The temporal fluctuations in fluorescence intensity observed in RhPh–actin filaments were investigated in this study by comparing various observation conditions to unveil the underlying mechanism. These fluctuations were observed when intense excitation light was applied in the presence of glucose and glucose oxidase (Fig. 7, *A*–*C*). The changes in fluorescence intensity were reversible, and each state persisted for several 100 ms. The possibility that this could be due to dissociation and rebinding of RhPh can be excluded because free RhPh was washed out of the flow cell before the observations.

Furthermore, the temporal fluctuations in fluorescence intensity were markedly reduced by the addition of Trolox (Fig. 7*E*). Such fluctuations typically arose under oxygen-scavenging conditions and were effectively suppressed by triplet-state quenchers like Trolox. The mechanisms may be explained by the photophysical property of fluorophores, as follows.

Upon excitation by light, most fluorophores transition from the ground state to the singlet excited state, from which they typically return to the ground state by emitting fluorescence. However, a small fraction of the excited molecules can undergo intersystem crossing to the triplet state, which is nonemissive and energetically less stable, resulting in a transient dark phase. These dark states, generally called “blinking,” typically have lifetimes on the order of microseconds (54, 55). Fluorophores in the triplet state readily react with molecular oxygen in solution, generating reactive oxygen species, which can lead to photobleaching of the dye and cause damage to samples such as actin filaments (56). To minimize these detrimental effects, oxygen scavenging systems such as glucose and glucose oxidase are commonly employed in fluorescence imaging (57).

However, molecular oxygen also acts as a quencher of the triplet-state fluorophores by facilitating their return to the ground state (58). Thus, oxygen scavenging systems may inadvertently prolong the occupancy of fluorophores in the nonemissive triplet state, and the resulting dark-state lifetimes can range from several to hundreds of milliseconds (52, 59). This timescale is consistent with the fluorescence intensity fluctuations observed in this study (Fig. 7*B*). Trolox is commonly added to imaging solutions as an alternative triplet-state quencher to molecular oxygen and has been shown to effectively suppress both photobleaching and blinking. This effect has been demonstrated in various fluorophores, including Cy5 and ATTO647N (51, 52). The mechanism involves the combined actions of Trolox and its oxidized form, Trolox quinone, which function respectively as a reducing and an oxidizing agent. Together, they facilitate rapid recovery of fluorophores from the triplet state to the ground state, thereby minimizing the time spent in nonemissive states (47, 48).

Given the photophysical properties of fluorophores described previously, it is reasonable to conclude that the substantial temporal fluctuations observed in RhPh–actin filaments were induced by the absence of molecular oxygen in the imaging solution because of the glucose and glucose oxidase system and were subsequently suppressed by triplet-state quenching through the addition of Trolox. Because such temporal fluctuations in fluorescence intensity of RhPh-labeled actin filaments potentially complicates quantitative fluorescence analyses, these findings provide a valuable experimental recommendation for minimizing artifacts in time-lapse fluorescence imaging of actin filaments and possibly other cytoskeletal structures. However, the mechanistic basis for how dozens of fluorophores within a small region along an actin filament exhibit synchronized temporal fluctuations remains unclear. This raises intriguing questions about the potential for cooperative photophysical behavior or underlying structural dynamics of the filament that could influence fluorophore states in a spatially coordinated manner.

## Experimental procedures

### Preparation and labeling of actin filaments

Actin was purified from rabbit skeletal muscle (60), rapidly frozen, and stored at −80 °C. This G-actin was thawed just before use and polymerized at 23 °C in F-buffer (10 mM Hepes–HCl [pH 7.4], 100 mM KCl, 2 mM MgCl_2_, 1 mM ATP, and 1 mM DTT) for 1 h. Actin filaments at various concentrations were labeled with 1 μM RhPh (Fujifilm Wako) or Alexa488Ph (Invitrogen) by incubating them in F-buffer at 23 °C in the dark for 15 min.

### Sample preparation for fluorescence microscopy observation

Surfaces of 22 × 32 mm glass coverslips (Matsunami Glass) used for observing actin filaments were cleaned by ultrasonic treatment in 8 M KOH for 1 h, followed by a thorough rinse with ultrapure water. The aminosilanization of the glass surface was then performed by incubating it in a solution of 3-aminopropyltriethoxysilane (Shin-Etsu Chemical) (diluted 1/10,000 in water) for 10 min. The coverslip was then thoroughly rinsed with ultrapure water, dried, and used within 1 week. The flow cell was constructed by combining the amino-silanized coverslip and an 18 × 18 mm glass coverslip (#1, Matsunami Glass) separated by two strips of double-sided adhesive tape (thickness: 0.09 mm), creating a volume of approximately 10 μl. The labeled actin filaments were introduced into the cell and allowed to bind to the glass surface by incubating for several minutes. Subsequently, the cell was washed with 50 μl of F-buffer to remove free fluorescent Ph in the solution. In some experiments, F-buffer containing the antibleaching reagents (1 mM Trolox, 3 mg/ml glucose, 0.2 mg/ml glucose oxidase, and 0.04 mg/ml catalase) was introduced into the cell just before observation.

The methylcellulose-containing observation cells were assembled similarly using cleaned plain 22 × 32 mm glass coverslips. To prevent nonspecific binding of actin or RhPh to the glass surface, the glass surface was initially coated with bovine serum albumin at 10 mg/ml in F-buffer. Subsequently, the labeled actin filament solution containing 0.4% methylcellulose (methyl cellulose 4000; Wako Pure Chemicals; catalog no.: 132-02152) was introduced into the cell.

### Fluorescence microscopy observation

We observed actin filaments labeled with RhPh or Alexa488Ph using an epifluorescence microscope (IX71; Olympus) and an objective lens (PlanApo 100×/1.40; Olympus). The excitation light was provided by a controllable white light source (SOLA Light Engine; Lumencor), and the fluorescence from Rh and Alexa488 was observed through their respective filter sets (Rh: U-MWIGA3, Olympus; Alexa488: U-MNIBA2, Olympus) and captured by an electron multiplying charge-coupled device camera (iXonX3; Andor). The images to capture the inhomogeneity of spatial fluorescence intensity were taken with an exposure time of 1000 ms, whereas fluctuations in temporal fluorescence intensity were observed with an exposure time of 100 ms. The obtained images consisted of 512 × 512 pixels, each with a size of 80 × 80 nm.

### Spectrofluorometer measurements

Rh-NHS (Thermo Scientific) and Alexa Fluor 488-NHS (Invitrogen) were dissolved in dimethyl sulfoxide at a concentration of 50 mM. Before measuring fluorescence intensity, they were diluted to 1 μM with either pure water or methanol. The solution was then placed in a 1.5 × 1.5 mm layer thickness Ultra-Micro-cuvette (Hellma), and the fluorescence spectrum was measured using a spectrofluorometer (FP-8500; JASCO). Rh was excited with 545 nm light, and the fluorescence spectrum was recorded in the 560 to 610 nm range. Alexa488 was excited with 494 nm light, and the fluorescence spectrum was recorded in the 505 to 555 nm range. For all conditions, measurements were performed three times. Considering the filter set used for fluorescence microscopy observation, the integrated fluorescence intensity values were calculated at 575 to 610 nm for Rh and at 510 to 555 nm for Alexa488, and the results were compared.

### Analysis of actin filament fluorescence

The fluorescence intensity of the actin filaments was analyzed using Fiji (National Institutes of Health). We first selected filaments longer than 2 μm and without sharp kinks in the microscopic images and analyzed regions that did not overlap with other filaments. The segments of the filaments were selected as a line width of 21 pixels centered on the actin filament and subjected to linearization processing. To eliminate the influence of background light, a region of the same size as each filament was selected from an area near the filament where no filaments were present, and the average intensity of this region was subtracted from the intensity of the filament. The fluorescence intensity at each filament position was calculated as the average value of five pixels with the highest intensities in the direction perpendicular to the filament axis. The fluorescence intensity at each point was then averaged along the filament. The average value along each filament was normalized to 1, and the fluorescence intensity along the filament was then plotted on a graph. In addition, the average and SD of SDs of the normalized fluorescence intensity along each filament was obtained to quantitatively evaluate the spatial inhomogeneity of fluorescence intensity in the actin filaments. The kymographs were created using Fiji's Montage and pseudocolored using mpl-viridis from the lookup tables.

### Simulation of random binding

We simulated the fluorescence intensity of RhPh along an actin filament of 75 pixels, assuming random binding of RhPh molecules to actin protomers. Each pixel was 80 nm long and contained 32 actin protomers, so the total length of the filament was 6 μm (75 pixels). The random binding of RhPh to each actin protomer was simulated using the “random.choice” function in the Python “numpy” package. The binding probability of RhPh was set at the average binding density of RhPh based on the observation results. Next, we calculated the number of RhPh molecules present in each pixel and determined the fluorescence intensity of each pixel ($I_{j}$) based on the formula noted later, considering the influence of fluorescence spreading to neighboring pixels. We fitted the spatial spread of fluorescence in the direction perpendicular to the filament axis of the observed actin filaments with a Gaussian distribution, and σ was determined to be 145 nm. Finally, the fluorescence intensity was normalized to have a mean value of 1 and graphed. The fluorescence image of the filaments obtained through simulation was also visualized using the Python PIL package, considering similar spatial spreading in the direction perpendicular to the filament axis.

$$\begin{array}{c} I_{j}=\sum_{i=1}^{75}\frac{n_{i}}{\sqrt{2\pi {\left(\frac{\sigma }{80}\right)}^{2}}}\exp\left(-\;\frac{{\left(i\;-\;j\right)}^{2}}{2{\left(\frac{\sigma }{80}\right)}^{2}}\right)\;\left(1\;\leq \;j\;\leq \;75\right) \end{array}$$

## Data availability

Data will be shared upon request to T. Q. P. U. (t-uyeda@waseda.jp).

## Supporting information

This article contains supporting information.

## Conflict of interest

The authors declare that they have no conflicts of interest with the contents of this article.
