# Supplementary material for: Spatiotemporal fluctuations in fluorescence intensity of rhodamine phalloidin–labeled actin filaments
Source: J Biol Chem. 2025 Jun 24;301(8):110417. doi: 10.1016/j.jbc.2025.110417 (PMC12305204; doi:10.1016/j.jbc.2025.110417)
Supplement: Supporting Information [file mmc1.pdf]

Supporting Information

Spatiotemporal fluctuations in fluorescence intensity of rhodamine phalloidin-labeled actin filaments

Kenta Toshino[1], Yosuke Yamazaki[1,§], Shunsuke Ando[1], Ryuichi Kaneda[1], Kazunori Ono[1], Takahiro Suzuki[1], Saku T. Kijima[2,¶], Taro Q.P. Uyeda[1,2,*]

1: Department of Pure and Applied Physics, Graduate School of Advanced Science and Engineering, Waseda University, Shinjuku, Tokyo 169-8555, Japan
2: Biomedical Research Institute, National Institute of Advanced Industrial Science and Technology, Ibaraki 305-8565, Japan.
§ Present address: RIKEN Center for Biosystems Dynamics Research, Kanagawa 230-0045, Japan.
¶ Present address: Bioproduction Research Institute, National Institute of Advanced Industrial Science and Technology, Ibaraki 305-8566, Japan.
[*] Correspondence: t-uyeda@waseda.jp

Exclusion of the effect of denatured actin on the spatial fluorescence inhomogeneity of RhPh-actin filaments

The G-actin purified from rabbit skeletal muscle used in this study was flash-frozen in liquid nitrogen and stored at –80°C. Prior to polymerization, it was thawed and used directly. However, such freeze–thaw cycles carry the potential risk of partial denaturation of actin molecules. If partially denatured actin is incorporated into filaments, it may affect the Ph-binding site, thereby locally reducing or enhancing the fluorescence intensity and potentially contributing to spatial inhomogeneities in RhPh fluorescence.

To test this possibility, we prepared filaments from actin that had undergone a full polymerization–depolymerization cycle to ensure the exclusion of defective monomers. Thawed actin was first dialyzed overnight against G-buffer (2 mM Tris-HCl pH 8.0, 0.2 mM $CaCl_2$, 0.005% NaN_3, 0.2 mM ATP, 1 mM DTT), and the supernatant was collected after ultracentrifugation (80,000 rpm for 30 min at 5 ℃). This actin was polymerized in F-buffer (10 mM HEPES-HCl pH 7.4, 100 mM KCl, 2 mM $MgCl_2$, 1 mM ATP, 1 mM DTT) for 2 h at room temperature and pelleted again by ultracentrifugation. The pellet was resuspended in G-buffer and dialyzed overnight to depolymerize the filaments. A final ultracentrifugation was performed, and the supernatant containing cycled G-actin

was collected. The actin concentration was determined by absorbance at 290 nm and used for subsequent experiments.

Even when 1/8xRhPh-labeled actin filaments were prepared from this cycled actin, they still exhibited spatial fluorescence inhomogeneity (Fig.S1*A, B*). The average fluorescence intensity was 953 ± 163 a.u. (n = 18 filaments), and the average standard deviation (SD) of the normalized fluorescence intensity along the filaments was 0.243 ± 0.059 (n = 18 filaments). This SD was not significantly different from that of filaments prepared from actin that did not undergo cycling, shown in the main text (Fig.S1*C*).

These results demonstrate that the spatial inhomogeneity observed in 1/8xRhPh-actin filaments is not an artifact arising from freeze–thaw-induced denaturation of actin. Instead, it likely reflects an intrinsic property of actin filaments, particularly their structural polymorphism.

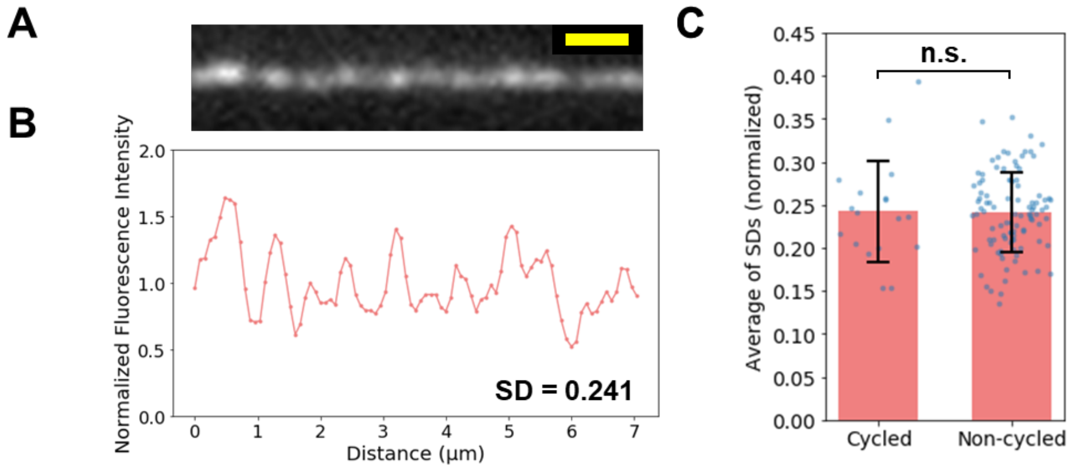

Figure S1. Effects of additional polymerization-depolymerization cycle on spatial fluorescence inhomogeneity. *A*, a representative fluorescence image of a 1/8xRhPh-labeled actin filament prepared from cycled G-actin. *B*, intensity distribution along the filament shown in *A*. *C*, comparison of the average of SDs of the normalized fluorescence intensity along the length of filaments prepared from cycled G-actin (n = 18 filaments) and non-cycled G-actin (n = 100 filaments). The data of 1/8xRhPh-actin filaments from non-cycled G-actin is reproduced from Figure 5*C* for comparison. Each data point represents the SD of fluorescence intensity along one actin filament. The observed data of filaments prepared from cycled G-actin were obtained by analyzing filaments in 6 images taken in two independent experiments.

Effect of ionic strength on the spatial inhomogeneity of fluorescence intensity in RhPh-labeled actin filaments

To determine whether the increased ionic strength is involved in the Pi-induced reduction in the average of SDs of normalized fluorescence intensity and the decreased correlation between RhPh and Alexa488Ph, as reported in the main text and Fig. 6, we conducted a control experiment using 20 mM K_2SO_4 instead of 20 mM KH_2PO_4/K_2HPO_4. Actin filaments were incubated with 20 mM K_2SO_4 for 10 minutes prior to labeling with 1/8xRhPh and imaged under the same conditions. The observed fluorescence intensity was 1304 ± 256 a.u., corresponding to a labeling ratio of 12.8%, which was comparable to that observed in the presence of 20 mM KH_2PO_4/K_2HPO_4 (13.4%). However, the average of SDs of normalized fluorescence intensity along the filaments was significantly higher under the K_2SO_4 condition (0.247 ± 0.045) than in the presence of KH_2PO_4/K_2HPO_4. Additionally, this value was significantly greater than the SD obtained from simulations assuming random RhPh binding at the same 12.8% labeling ratio (0.192 ± 0.038). Furthermore, co-labeling of actin filaments with RhPh and Alexa488Ph in the presence of 20 mM K_2SO_4 yielded a strong correlation between the two fluorescence intensities ($r = 0.75$). These results indicate that the reduction in SD and correlation observed in the presence of KH_2PO_4/K_2HPO_4 is not attributable to increased

ionic strength, but rather to a specific effect of Pi on the structural polymorphism of the actin filaments.

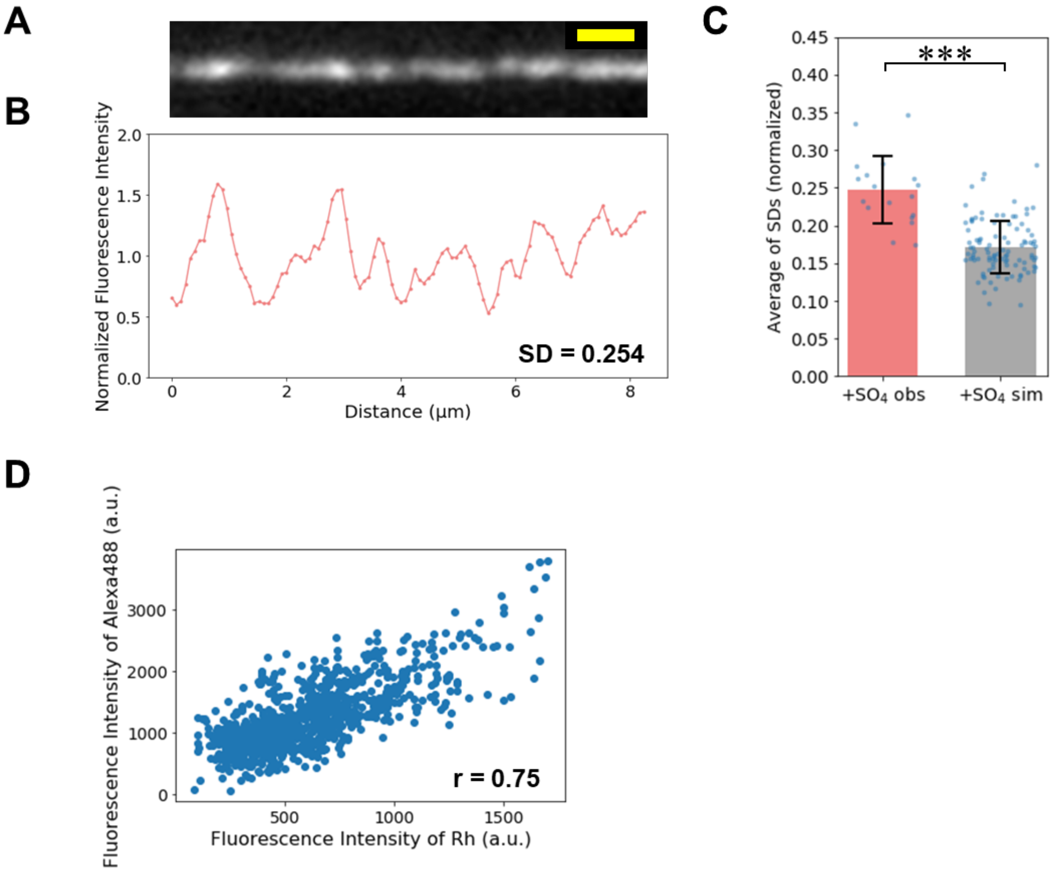

Figure S2. Spatial inhomogeneity of fluorescence intensity in RhPh-labeled actin filaments in the presence of K_2SO_4. *A*, a representative fluorescence image of 1/8xRhPh-actin filaments incubated in the presence of 20 mM K_2SO_4. *B*, normalized fluorescence intensity distribution along the filament shown in *A*. *C*, comparison of the average of SDs of the normalized observed fluorescence intensities along 1/8xRhPh-labeled filaments under the K_2SO_4 condition (n = 18 filaments), and simulations at 12.8% binding density

(n = 100 filaments). *** indicates $p < 0.001$ (Welch's t-test). Each point represents the SD of fluorescence intensity along one actin filament. The observed data were obtained by analyzing filaments in 6 images taken in two independent experiments. For the simulation data, 100 independent simulations were performed, and the SD calculated from each simulation was plotted as an individual data point. *D*, a scatter plot of the Rh and Alexa488 fluorescence intensities at each pixel in actin filaments co-labeled with 1/8xRhPh and 1/8xAlexa488Ph in the presence of 20 mM K_2SO_4. Total of 941 pixels along 9 actin filaments were analyzed.

Effects of divalent cations on the spatial inhomogeneity of fluorescence intensity in RhPh-labeled actin filaments

In the experiments shown in the main text of this paper, Ca^{2+}-bound G-actin was polymerized in the presence of Mg^{2+}, without chelation of Ca^{2+} prior to polymerization, raising the possibility that both Mg^{2+}- and Ca^{2+}-bound actin protomers were incorporated into the same filament. To examine if the bound divalent cation influences the spatial inhomogeneity of RhPh fluorescence, we prepared actin filaments bound exclusively with either Mg^{2+} or Ca^{2+}, and compared the SD of normalized fluorescence intensity as well as the correlation between RhPh and Alexa488Ph fluorescence.

Actin used in this supplementary experiment was dialyzed overnight against G-buffer and clarified by ultracentrifugation (80,000 rpm x 30 min at 5 °C) before polymerization. The concentration of Ca^{2+}-bound G-actin in the supernatant after ultracentrifugation was determined by absorbance at 290 nm. For the preparation of Mg^{2+}-actin filaments, Ca^{2+}-bound G-actin in G-buffer was incubated with 1 mM EGTA and 50 μM $MgCl_2$ on ice for at least 10 min to exchange the bound cation. Polymerization was carried out in F-buffer. For the preparation of Ca^{2+}-actin filaments, Ca^{2+}-bound G-actin in G-buffer was polymerized in Ca-F-buffer that contained 2 mM $CaCl_2$ in place of $MgCl_2$, followed by incubation at room temperature for at least 2 h.

The average fluorescence intensity of Mg^{2+}-actin filaments labeled with 1/8x RhPh, was 1143 ± 224 a.u. (n = 18 filaments), and the SD of normalized fluorescence intensity was 0.269 ± 0.056 (Fig. S3*A, B*). This SD value was significantly greater than that obtained from simulations assuming random RhPh binding at the corresponding labeling density (11.2%), indicating the presence of additional sources of fluorescence inhomogeneity beyond random binding (Fig. S3*C*). Moreover, filaments co-labeled with RhPh and Alexa488Ph exhibited a positive correlation (r = 0.66) between the two fluorescence intensities, consistent with a non-random spatial distribution of Ph binding (Fig. S3*D*).

Similarly, in Ca^{2+}-actin filaments labeled with 1/8x RhPh, the average fluorescence intensity was 1065 ± 184 a.u., and the SD of normalized fluorescence intensity was 0.247 ± 0.040 (n = 18 filaments) (Fig.S4*A, B*). This value also exceeded the SD predicted by random labeling based on the calculated labeling density (10.5%) (Fig. S4*C*). The correlation coefficient between RhPh and Alexa488Ph fluorescence intensities in Ca^{2+}-actin filaments was 0.43, again supporting the presence of regionally biased Ph binding (Fig. S4*D*).

Taken together, these results confirm that the spatial fluorescence inhomogeneity and positive RhPh–Alexa488Ph correlation reported in the main text are not attributable to

mixed populations of Ca^{2+}- and Mg^{2+}-bound actin protomers. Instead, they reflect intrinsic structural properties of actin filaments that are independent of the identity of the bound divalent cation.

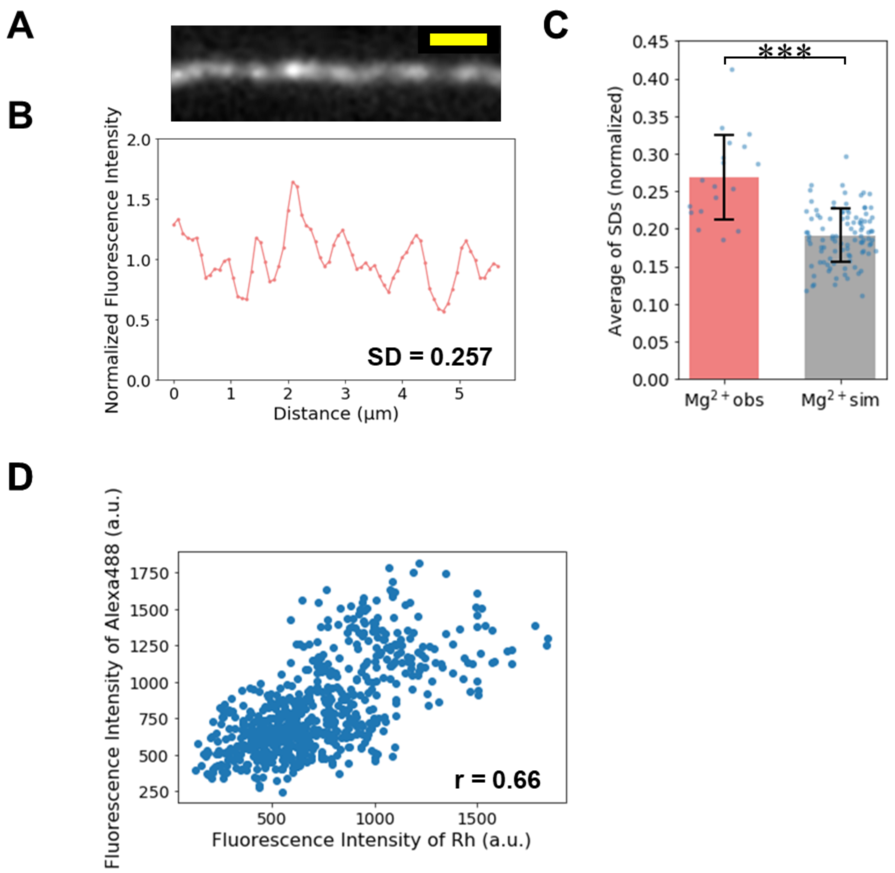

Figure S3. Spatial inhomogeneity of fluorescence intensity along RhPh-labeled Mg^{2+}-actin filaments. *A*, a representative fluorescence image of 1/8xRhPh-Mg^{2+}-actin filament. *B*, normalized fluorescence intensity distribution along the filament shown in *A*. *C*, comparison of the average of SDs along the length of the normalized observed fluorescence intensities of 1/8xRhPh-Mg^{2+}-actin filaments (n = 18 filaments), and

simulations at 11.2% binding density (n = 100 filaments), calculated based on the average fluorescence intensity of the observed 18 filaments (1143 ± 224 a.u.). *** indicates $p < 0.001$ (Welch's t-test). Each data point represents the SD of fluorescence intensity along one actin filament. The observed data were obtained by analyzing filaments in 6 images taken in two independent experiments. For the simulation data, 100 independent simulations were performed, and the SD calculated from each simulation was plotted as an individual data point. *D*, a scatter plot of the Rh and Alexa488 fluorescence intensities at each pixel in 1/8xRhPh-1/8xAlexa488Ph Mg^{2+}-actin filaments. Total of 711 pixels along 9 actin filaments were analyzed.

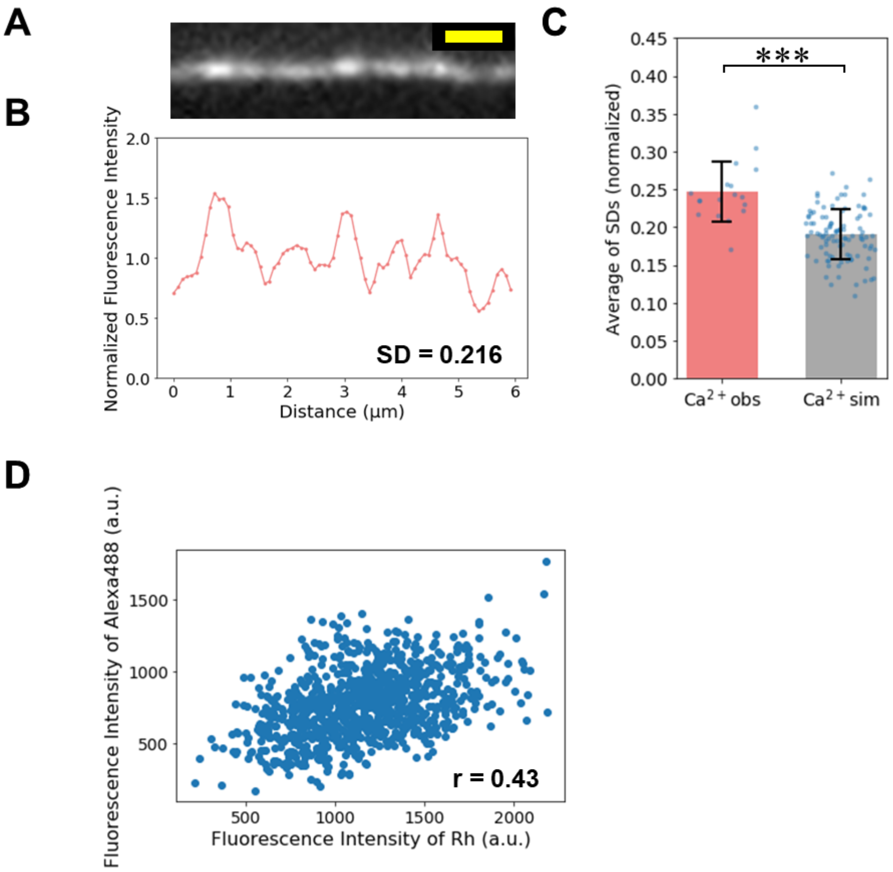

Figure S4. Spatial inhomogeneity of fluorescence intensity along RhPh-labeled Ca^{2+}-actin filaments. *A*, a representative fluorescence image of 1/8xRhPh-Ca^{2+}-actin filament. *B*, normalized fluorescence intensity distribution along the filament shown in *A*. *C*, comparison of the average of SDs along the length of the normalized observed fluorescence intensities of 1/8xRhPh-Ca^{2+}-actin filaments (n = 18 filaments), and simulations at 10.5% binding density (n = 100 filaments), calculated based on the average fluorescence intensity of the observed 18 filaments (1065 ± 184 a.u.). * indicates $p < 0.001$ (Welch's t-test). Each data point represents the SD of fluorescence intensity along one actin filament. The observed data were obtained by analyzing filaments in 6 images

taken in two independent experiments. For the simulation data, 100 independent simulations were performed, and the SD calculated from each simulation was plotted as an individual data point. *D*, a scatter plot of the Rh and Alexa488 fluorescence intensities at each pixel in 1/8xRhPh-1/8xAlexa488Ph Ca^{2+}-actin filaments. Total of 952 pixels along 9 actin filaments were analyzed.

Video S1. 1/8xRhPh-actin filaments observed using a methylcellulose observation chamber. The filaments were observed in F-buffer containing 0.4% methylcellulose (without Trolox, glucose oxidase, or glucose). Time-lapse observations were conducted with successive 100 ms exposures for 6 s. The movie shows 7 frames per second.

Video S2. Temporal fluctuations in fluorescence intensity in 1/8xRhPh-actin filaments. The filaments were observed in F-buffer containing the anti-bleaching reagents (3 mg/mL glucose, 0.2 mg/mL glucose oxidase, 0.04 mg/mL catalase). Time-lapse observations were conducted with successive 100 ms exposures for 6 s at 80% excitation light intensity. The movie shows 7 frames per second.
